# Evaluation of HIV-Related Cardiomyopathy in HIV-Positive Patients in Bushehr, Iran

**DOI:** 10.7759/cureus.28078

**Published:** 2022-08-16

**Authors:** Farhad Abbasi, Asha Alexander, Soolmaz Korooni Fardkhani, Dariush Iranpour, Kamran Mirzaei, Mohammadreza Kalantarhormozi, Mehrdad Haghighi, Marziyeh Bagheri

**Affiliations:** 1 Infectious Diseases, Bushehr University of Medical Sciences, Bushehr, IRN; 2 Medical Research, Nanjing Medical University, Nanjing, CHN; 3 Internal Medicine, Bushehr University of Medical Sciences, Bushehr, IRN; 4 Cardiology, Bushehr University of Medical Sciences, Bushehr, IRN; 5 Community Medicine, Bushehr University of Medical Sciences, Bushehr, IRN; 6 Endocrinology, Bushehr University of Medical Sciences, Bushehr, IRN; 7 Infectious Diseases and Tropical Medicine Research Center, Shahid Beheshti University of Medical Sciences, Tehran, IRN; 8 Medical Research, Bushehr University of Medical Sciences, Bushehr, IRN

**Keywords:** cardiovascular disease, aids, hiv, hiv cardiomyopathy, cardiomyopathy

## Abstract

Objectives

In 2020, according to the UNAIDS (Joint United Nations Programme on HIV/AIDS), more than 37 million people lived with human immunodeficiency virus (HIV) infection worldwide. The disease is known to affect several organs, and one of the most affected organs is the heart. Cardiac diseases are highly prevalent among HIV-infected individuals, and recent findings suggest that this could be due to the damage caused by the virus. HIV patients are subject to advanced immunosuppression, which may lead to cardiac muscle damage and, in turn, cardiomyopathy. We aimed to study the incidence of HIV-related cardiomyopathy.

Methods

A pilot cross-sectional study was conducted to assess cardiomyopathy among 200 HIV patients who presented to the Heart Center, Bushehr, Iran. Patients’ files were used to determine the demographic data including age, gender, education, marital status, history of illicit drug use, unsafe/unprotected sexual contact, and whether the patient was a prisoner. Several laboratory data were also collected from these files. Physical examination of the cardiovascular system and echocardiography were also included as part of the evaluation.

Results

Although at least four out of five patients presented with some kind of cardiac damage, including valvular damage and pericardial effusion, none was diagnosed with cardiomyopathy. Valvular dysfunction was detected in 88.5% of the patients. Diastolic dysfunction was found in 7.7% of them. The mean ejection fraction was found to be 58%. In addition to cardiomyopathy, none of the patients developed systolic dysfunction, wall motion abnormality, intra-cardiac mass, or vegetation.

Conclusions

Cardiovascular complications are common among HIV-infected patients. Cardiomyopathy was not detected in our patients. In addition, the most common manifestations that were detected among our patients were valvular heart diseases and pericardial effusion.

## Introduction

According to the UNAIDS (Joint United Nations Programme on HIV/AIDS), more than 37 million people were living with human immunodeficiency virus (HIV) infection worldwide by the end of 2020. Of these, at least 1.5 million people were infected in the same year. HIV patients are likely to develop cardiovascular diseases potentially due to the direct effects of the virus [1,2]. The disease is known to affect several organs including the cardiovascular system, the hematopoietic system, the nervous system, the gastrointestinal tract, and the genitourinary system [3]. One of the most affected organs is the heart. Opportunistic infections and direct invasion of the virus in different tissues could further complicate the acquired immunodeficiency syndrome.

Cardiac diseases lead to an increase in the mortality rate in the general population. A similar trend has also been observed among HIV patients. It is predicted that at least 6.5% of the HIV infected could die due to an underlying cardiovascular disease [4]. Sudden cardiac death can be associated with increased viral load and low CD4 count among these individuals [5].

HIV-induced immunosuppression and opportunistic infections have also been found to cause cardiovascular diseases, particularly in patients from developing countries where access to antiretroviral therapy (ART) medications could be a challenge. This is particularly true in the low- and middle-income countries, where the HIV-1 epidemic has succeeded in infecting a major population due to its high degree of viral multiplication [6,7]. In the developed world, the opposite scenario exists. Due to the easy availability of ART, the ill effects of these drugs lead to several cardiac diseases. ART could lead to lipodystrophy, lipoatrophy, and dyslipidemia [8]. The newer highly active ART (HAART) has increased the lifespan of patients and hence they more prone to heart diseases. The likelihood of cardiac ailments among HIV patients has increased by around 50% [2]. The most common cardiac diseases affecting these patients include dilated cardiomyopathy, coronary artery disease, pericardial effusion, and pulmonary hypertension. HIV can therefore be viewed as a potential risk factor for coronary artery disease, and the dilemma facing clinicians is how to quantify this risk.

HIV infection leads to myocardial damage, which may be due to the virus, the toxins produced by it, the nutritional status of the patient, and the autoimmunity of the disease. Dilated cardiomyopathy that occurs in HIV-infected patients poses diagnostic and therapeutic challenging problems [9]. Left ventricular (LV) dysfunction as a result of myocardial diseases is an independent risk factor for death. It tends to increase the risk of death independent of the CD4 cell count and HIV viral load [10]. One of the major issues faced by clinicians is that the majority of the cardiac abnormalities in HIV-infected patients remain undetected or misdiagnosed mostly due to their atypical presentation [11].

At the moment, there is no specific treatment regimen devised to improve the prognosis of HIV patients with cardiac diseases. It is suggested that these patients should be subject to lifestyle modifications to reduce the risk of cardiac diseases. Antiretroviral drugs should also be carefully selected according to the cardiovascular risk factors in this population [12]. However, studies involving larger populations are required to provide better guidelines for the management of these diseases [13].

Cardiovascular diseases greatly impact HIV-infected individuals when compared to non-infected individuals of the same age. The most likely causes of cardiovascular diseases among HIV patients are cardiomyopathy, accelerated atherosclerosis, pulmonary arterial hypertension, vasculitis, myocarditis, pericardial diseases, and endocarditis [14]. The cause of cardiomyopathy in patients infected with HIV remains largely unknown, although several predisposing factors have been identified.

Myocardial invasion of HIV tends to increase the local inflammatory cytokines, thus leading to an influx of lymphocytic B cells into the myocardium [15]. Several cardiac muscle-specific antibodies have also been identified among cardiac patients with HIV [16]. Additionally, nutritional deficiencies are common in HIV infection. These include the deficiency of selenium, vitamin B12, carnitine, growth hormones, and thyroid hormones, most likely in the late stages of the disease. Severe deficiencies of these nutrients are associated with LV dysfunction [8]. Similarly, malnutrition can also be a contributory factor. A lower body mass index is associated with cardiomyopathy in people who are living with HIV [17]. Hyperlipidemia, hyperglycemia, and lipodystrophy are a few side effects observed when patients are under HAART, particularly protease inhibitors [18]. Furthermore, prolonged immunosuppression coupled with the above effects of the virus may cause cardiac complications [13].

We devised our research to study the incidence of cardiomyopathy among HIV patients. Ejection fraction (EF), valvular/systolic/diastolic dysfunction, wall motion abnormality, intracardiac mass, and pericardial effusion were considered to evaluate the effect of HIV on the heart muscles.

## Materials and methods

Eligibility criteria

An institution-based pilot cross-sectional study was conducted to assess the incidence of cardiomyopathy among HIV patients who were HIV seropositive in the Heart Center Hospital, Bushehr, Iran, for over a year. The source population comprised all HIV patients who had been on follow-up at the unit for management of the heart condition. Around 200 clinical files of patients were evaluated for this pilot intervention study. Sample size estimation was not required as this was a study to start empiric treatment for cardiomyopathy in HIV patients.

Study variables

Independent variables such as sociodemographic factors, duration of the HIV infection, laboratory data, echocardiography study, and use of ART were collected from the patients’ files. The sociodemographic data comprised age (in years), gender, marital status, educational level, substance abuse, the practice of safe sex, and prison history. Data of blood investigations such as complete blood count (CBC), white blood count (WBC), fasting blood sugar (FBS), blood urea nitrogen (BUN), creatinine (Cr), alanine aminotransferase (ALT), aspartate transaminase (AST), alkaline phosphatase (ALP), cholesterol, triglycerides (TG), erythrocyte sedimentation rate (ESR), C-reactive protein (CRP), and CD4 count were also documented. Echocardiography was performed by our cardiologist at the center was reviewed, and information about the EF, heart valvular dysfunction, cardiomyopathy, and its type (dilated, hypertrophic, ischemic), systolic dysfunction, diastolic dysfunction, wall motion abnormality, vegetation, intracardiac mass, and pericardial effusion in these patients were gathered.

Data tools, collection, and quality control

Initially, patient records were reviewed for information. The data abstraction tool was then developed based on the observations, and a plan was devised to segregate the required data. The research project was financially supported by the office of the Vice-Chancellor for Research at the Bushehr University of Medical Sciences. The members of the faculty of medicine at the university were involved in data collection and evaluation. The data were then checked by the supervisor of the same department. The completeness of the information in the questionnaires was checked thoroughly and, any gaps identified were immediately rectified.

## Results

Sociodemographic factors

Of the 200 patients, 160 (80%) patients were male and the remaining 40 (20%) were female. The patients had a mean age of 37.9 ± 10.8 years, with an age range of 11 to 70 years. Around 57% were married, 31% were single, 9.5% were divorced, and 5% were undetermined. Around 30% of the patients were not educated, whereas 40.5% had elementary school education, 17.5% had guidance school education, 1.5% had a diploma, and 10.5% of the patient’s educational status was undetermined.

At least 64% of the patients had a history of illicit intravenous drug use (IDU), 33.5% had no history of IDU, and 2.5% had an unknown status. Evaluation of sexual contact revealed that 46.5% of patients had unsafe and unprotected sex, whereas 50.5% of them did not; 3% had an unknown status. A look at their prison history revealed that 65% of patients had been a prisoner at some point in their life and 30% had not, with 5% of them having declined to disclose this information.

Laboratory data

As shown in Table 1, laboratory analysis showed that all patients had WBCs between 5,347 ± 1,942 cells/mm^3^, an average Hb of 12.75 g/dL, and platelets between 192,000 ± 78,000/mm^3^. ALT and ALP had a mean of 41 mg/dL and 49 mg/dL, respectively. The former ranged from 3 mg/dL to 420 mg/dL, whereas the latter ranged from 3 mg/dL to 393 mg/dL. Similarly, ALP also had a huge range of values ranging from 28 mg/dL to 1,009 mg/dL, with an average of 255 mg/dL. Cholesterol ranged from 71 mg/dL to 1,555 mg/dL, with a mean of 389 mg/dL. The mean CD4 count of our patients was 389 ± 316 cells/mm^3^, with a minimum of 7 cells/mm^3^ and a maximum of 1,739 cells/mm^3^. The median CD4 count was 328/mm^3^.

**Table 1:** Laboratory analysis data of patients who participated in the study FBS, fasting blood sugar; BUN, blood urea nitrogen; Cr, creatinine ; ALT, alanine aminotransferase; AST, aspartate aminotransferase; ALP, alkaline phosphatase; TG, triglyceride

Variable	Mean	Standard Deviation	Minimum	Maximum
WBC(/mm^3^)	5347	194	1800	12700
HB(mg/dL)	12.7	2.0	7.9	18.0
Platelet(/mm^3^)	192,533	78,650	16,000	720,000
CD4 count (/mm^3^)	389	316	7	1,739
FBS (mg/dL)	94	27	6	1,739
BUN (mg/dL)	13	6	5	297
Cr (mg/dL)	1.3	0.3	0.5	3.3
ALT (mg/dL)	41	51	3	420
AST (mg/dL)	49	50	3	393
ALP (mg/dL)	255	141	28	1,009
TG (mg/dL)	127	61	16	499
cholesterol	162	118	71	1,555

The average of the duration of the HIV infection in our subjects was 5.4 years. Around 57% were complaint with ART with an average of 3.7 years, ranging from 2.1 to 5.3 years. The most common regimens were zidovudine + lamivudine + efavirenz and zidovudine + lamivudine + nevirapine. Echocardiography performed on all the 200 patients, which showed that mean EF of our patients was 58% + 3% with a range of 50-60%. Evaluation of echocardiography demonstrated that 11.5% had an average EF = 50%, 3.8% had EF = 55%, and 84.6% had EF = 60%. Valvular dysfunction was detected in 88.5% of our patients (categorized in Table 2). Our study found diastolic dysfunction in 7.7% of the patients. Although majority of patients had valvular dysfunction, none of the resultant patients had systolic dysfunction, wall motion abnormality, intracardiac mass, or vegetation. We detected pericardial effusion in 11.5% of our patients. None of our patients had cardiomyopathy.

**Table 2:** Frequency of valvular dysfunction in HIV-positive patients who participated in our study MR, mitral regurgitation; MVP, mitral valve prolapsed; TR, tricuspid regurgitation

Variable	Frequency	Percent (%)
Mild MR	5	21.7
Severe MVP	1	4.3
Mild TR	6	26.1
Mild MR + mild TR	7	30.4
Mild MR + mild MVP	1	4.3
Trivial MR + mild MVP	1	4.3
Mild MR + mild TR + mild MVP	2	8.7

## Discussion

HIV-infected patients have an increased propensity to develop heart diseases when compared to their non-infected counterparts. The most prevalent heart diseases in these individuals include myocardial infarction, cardiomyopathy, heart failure, and arrhythmia. The mortality in this population is also higher by at least 14% than among the general population [19]. Additionally, sudden cardiac death is ranked among one of the most common causes of death in HIV-infected patients [1]. The occurrence of cardiac death among such patients could be as high as 47% [20]. The hazard ratio for death with cardiomyopathy is about 4.0 and hence is extremely important to be ruled out of diagnosis [21].

Cardiomyopathy is a late-stage sequela of HIV infection. The virus tends to affect the entire heart, thus leading to structural dysfunctions [22]. HIV-mediated cytokine dysregulation may lead to an increase in tumor necrosis factor-alpha (TNF-alpha), interleukin-1 (IL-1), and IL-6, and thus be an important contributor to the development of HIV cardiomyopathy [23,24]. Several studies have detected cardiomyopathies, most commonly dilated cardiomyopathy among patients with HIV [8]. Chronic HIV infection can lead to chronic immune activation and inflammatory cytokine dysregulation. Myocarditis associated with the disease can lead to systolic and diastolic dysfunction. Right ventricular dysfunction is of particular interest since it is known to be associated with worse outcomes [25].

The HIV virus could directly invade and disrupt the myocardial structure. Co-infection with other pathogens can lead to severe damage to the myocardium due to its cumulative effect. Immunosuppression, viremia, and malnutrition caused by HIV infection could also cause myocardial damage [26]. Significant cardiac and skeletal muscle complications can occur independently of viral infection or replication due to HIV-1-related proteins. These proteins not only play the role of disease markers but also have significant biological activity that may lead to increased oxidative stress, stimulation of redox-sensitive pathways, and altered muscle morphologies. The use of dietary thiol supplements has been postulated to reduce skeletal and cardiac muscle dysfunction in HIV-1-infected individuals [27]. The impact of the virus may be minuscule in the form of microscopic inflammation seen only during autopsies or may cause severe loss of cardiac function and thus severe clinical outcomes. Irrespective of the pathogenesis, the disease is usually associated with a poor prognosis. At least 5% of cardiac HIV patients end up with heart failure.

At least a 30% reduction in the incidence of HIV-associated cardiomyopathy in the developed countries occurred mostly after protease inhibitor use. However, in developing countries, there is about a 32% increase in HIV-associated cardiomyopathy, which, in turn, has led to a high mortality rate for congestive heart failure [28]. In contrast, although HAART has dramatically reduced the morbidity and mortality associated with HIV infection, several antiretroviral toxicities have been described, including myocardial toxicity resulting from the use of nucleotide and nucleoside reverse transcriptase inhibitors (NRTIs). Current treatment guidelines recommend the use of HAART regimens containing two NRTIs for initial therapy of HIV-1 positive individuals [29]. A few mitochondrial-associated pathways of NRTI could lead to cardiomyopathy [30]. HAART includes protease inhibitors, which affect lipid and glucose metabolism and, in turn, may lead to premature vascular disease [31]. This may, in turn, lead to an iatrogenic metabolic syndrome called HIV-lipodystrophy syndrome. Young HIV patients have also been affected by this syndrome and later develop atherosclerotic diseases [32]. It is challenging, both diagnostically and therapeutically, when HIV/AIDS patients present with symptoms of cardiac diseases. Handling cardiac diseases in HIV/AIDS patients can vary significantly from non-HIV patients, mainly due to the presence of drug interactions, responsiveness, etc. [31].

Cardiomyopathy among HIV patients has been widely reported in the African subcontinent. In southern parts of Africa, vasculopathy associated with HIV has been recognized as the cause of high morbidity and mortality. Cardiomyopathy, pericarditis, and myocarditis are the most prevalent heart diseases among HIV patients in this region [33]. It has been theorized that patients of African descendants have a greater disposition to be infected with multiple cardiotropic viruses. Several viruses per case are associated with a greater viral burden in HIV-associated cardiomyopathy [15]. A co-infection of HIV/AIDS and Chagas disease commonly occurs in the tropics, which may present as severe cardiomyopathy [34].

In a study in Tanzania, around 10% of the patients presented with dilated cardiomyopathy [35]. In Romania, research on pediatric HIV-positive patients showed cardiovascular anomalies in 79 (67.52%) out of the 117 patients who were studied. Dilated cardiomyopathy was found in around 18.8% of cardiac patients [6].

Echocardiography was a useful technique for the early detection of cardiac dysfunction in asymptomatic HIV-positive carriers and AIDS patients. The frequency is related to the HIV infection stage and CD4+ counts. LV diastolic dysfunction can precede systolic dysfunction and may be a useful technique for the early detection of cardiac dysfunction [36].

On the other end of the spectrum, recent research shows that the incidence of cardiomyopathy among HIV patients has reduced by at least seven folds when under HAART as compared to their non-HAART counterparts. This is possible since HAART is known to prevent opportunistic infections and hence reduces the incidence of myocarditis [37].

Researchers from the United States of America found that fetuses exposed to HAART had better cardiac function up until two years of age [38]. Contradicting this, a US-based multicenter study found cardiomyopathies in the perinatal infected pediatric population even with ART. Nucleoside analogs, particularly zidovudine, were found to be associated with mitochondrial toxicity and thus lead to cardiomyopathy in children [39].

Cardiovascular abnormalities are common in HIV-infected patients; however, they can often be dormant [40]. Both structural and functional abnormalities are common. HIV cardiomyopathy has been postulated to be an important cause of heart failure and death [41]. Though noted to be important, a cross-sectional analysis in our paper showed several valvular dysfunction and no evidence of cardiomyopathy in our HIV-positive patients. Irrespectively, routine echocardiography is included in a patient’s standard care [42].

Careful selection of the antiretroviral drugs according to underlying cardiovascular risk factors is important. Cardiovascular risk reduction and lifestyle modifications also play an essential role in the proper management of these patients. This reiterated the theory put forth by several scientists throughout several decades, i.e., the need for cardiac evaluation among patients must be a priority [12].

In addition, it would have been possible to have a larger sample population and avoid potential selection bias if the study researchers had access to unregistered HIV patients. This could serve as a pilot study for an interventional study perhaps to justify why cardiomyopathy was not noted.

## Conclusions

Although cardiomyopathy is known for being an outcome of HIV, our pilot cross-sectional analysis did not detect cardiomyopathy among a sample size of 200 in the population of HIV-positive patients in Bushehr, Iran. Furthermore, irrespectively, there was no correlation between the duration of the HIV infection, the duration and compliance with ART, and the presence of cardiomyopathy in our population. An evaluation of 2,000 to 3,000 patients utilizing Iran's surveillance system of HIV data bank might be required to elicit the possible correlation.
